# Supplementary material for: High-throughput method characterizes hundreds of previously unknown antibiotic resistance mutations
Source: Nat Commun. 2025 Jan 17;16:780. doi: 10.1038/s41467-025-56050-2 (PMC11742677; doi:10.1038/s41467-025-56050-2)
Supplement: Supplementary file 1 — Supplementary Information [file 41467_2025_56050_MOESM1_ESM.pdf]

High-throughput method characterizes hundreds of novel antibiotic resistance mutations

Supplementary Information

Supplementary Figure 1. Mutations in *ykfM*.

Supplementary Figure 2. Mutations in *gyrB*.

Supplementary Figure 3. Mutation count in every condition.

Supplementary Figure 4. Sequencing depth.

Supplementary Figure 5. Secondary filtering criterion.

Supplementary Figure 6. Confirming mutations result from strong selective pressure.

Supplementary Figure 7. Validating resistance of Keio knockout strains.

Supplementary Table 1. Validating the number of mutations per genome by whole-genome sequencing individual clones.

Supplementary Table 2. Sequencing information for each sample.

Supplementary References

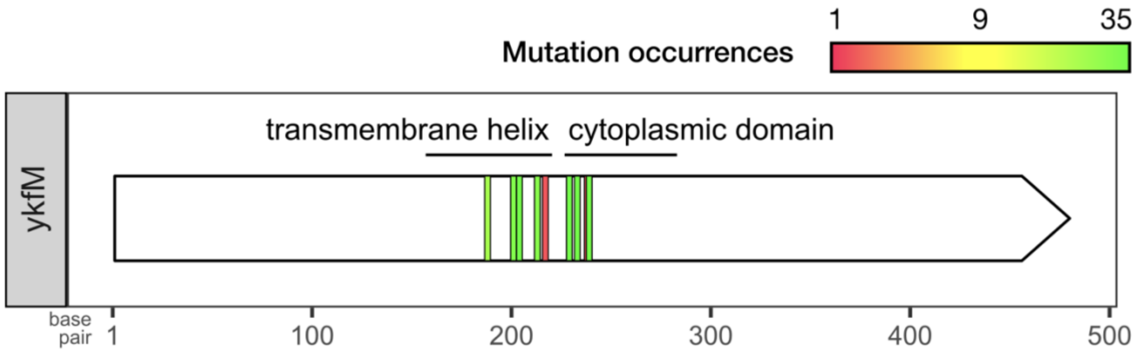

Supplementary Figure 1. Mutations in *ykfM*.

The *ykfM* gene sequence showing locations of computationally predicted domains, and the positions of mutations observed by QMS-seq. *ykfM* encodes an uncharacterized membrane protein – and had the most mutation occurrences of any gene in our dataset. The mutations clustered in its predicted transmembrane helix and cytoplasmic domains, suggesting it may be a transporter through which antibiotics can enter or exit the cell.

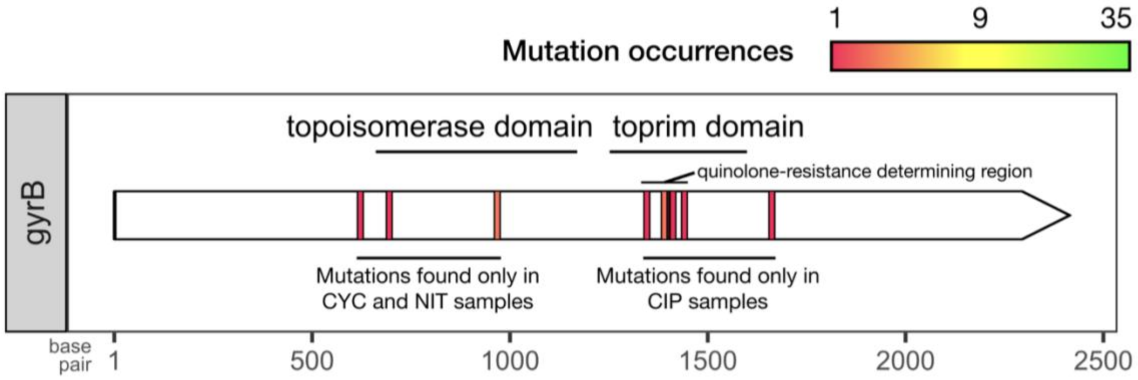

Supplementary Figure 2. Mutations in *gyrB*.

The *gyrB* gene sequence showing locations of known functional sites, and the positions of mutations observed by QMS-seq. GyrB is a secondary target for ciprofloxacin. Most of the mutations were specific to ciprofloxacin and clustered in or near the known 'quinolone-resistance determining region'. However, three other *gyrB* mutations occurred in cycloserine and nitrofurantoin samples, in a different region of the gene.

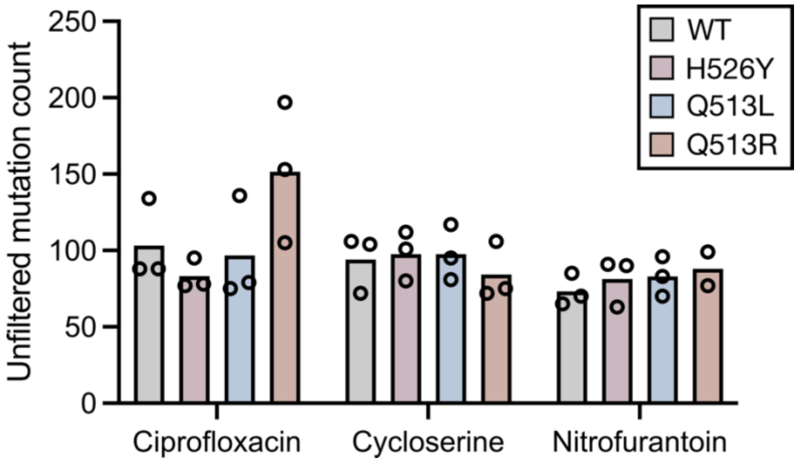

Supplementary Figure 3. Mutation count in every condition.
The average number of mutations identified in samples from the twelve different antibiotic / strain conditions. The value for each replicate sample is shown as a dot, bars are means (n=3).

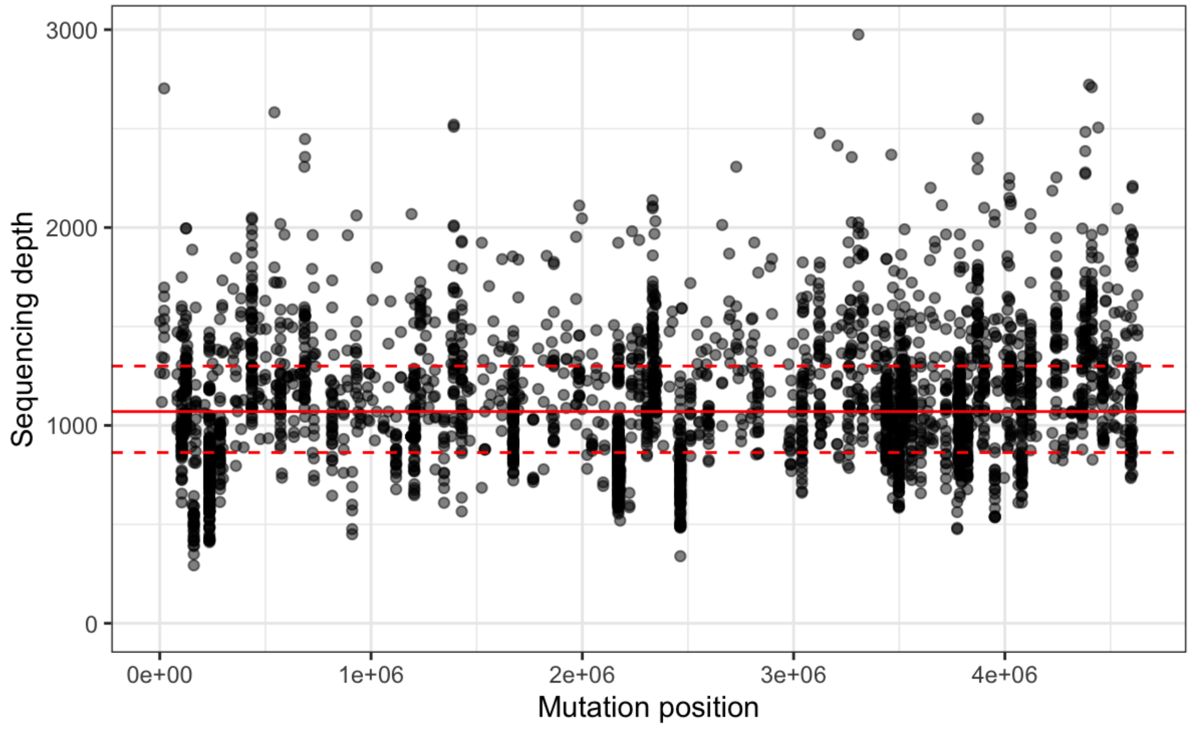

Supplementary Figure 4. Sequencing depth.

Sequencing depth at the loci of each of the 3,314 mutation occurrences. Solid red line is the median, dashed lines are upper and lower quartiles.

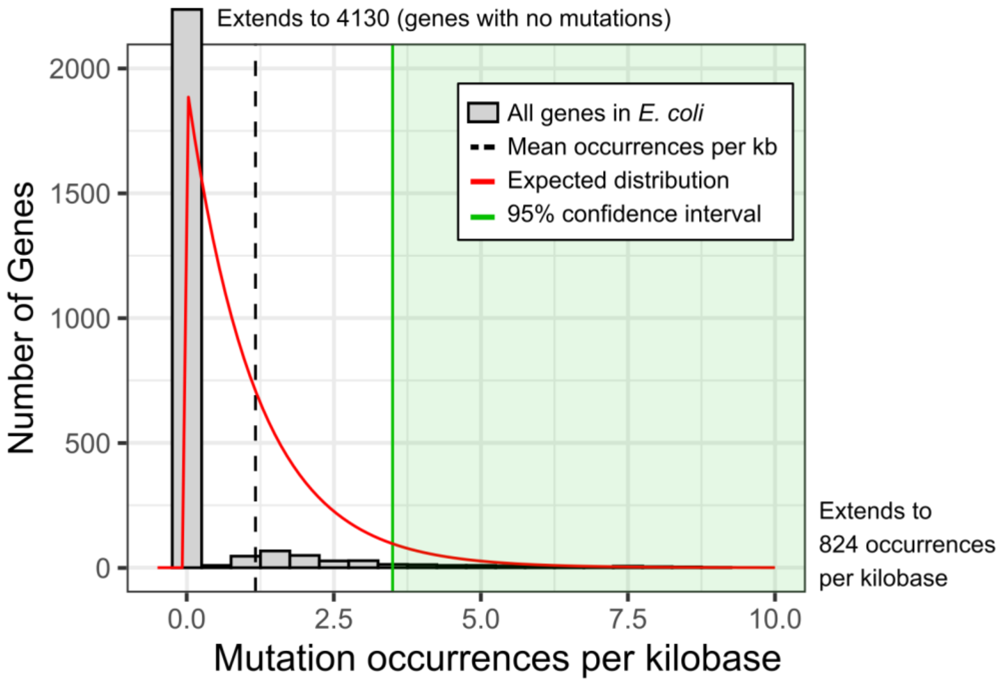

Supplementary Figure 5. Secondary filtering criterion.
In addition to our primary filtering criterion (mutations must appear across two or more independent samples) we also included single-sample mutations in genes where significantly more mutations were observed than expected. To determine this, we calculated the number of mutation occurrences per kilobase for every gene in *E. coli*. We then included single-sample mutations occurring in genes with more mutation occurrences per kilobase than the 95% confidence interval, equivalent to > 3.5 mutation occurrences / kb.

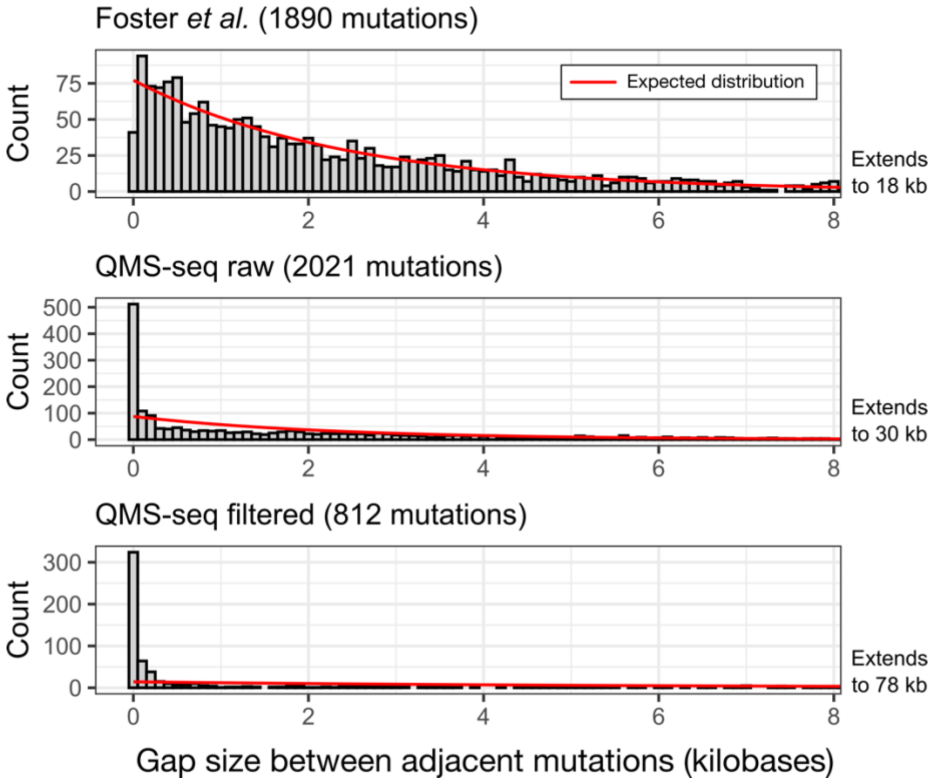

Supplementary Figure 6. Confirming mutations result from strong selective pressure. Comparing the distribution of mutations throughout the genome between QMS-seq and a mutation accumulation study performed in the absence of selective pressure using a Δ*mutL* strain of *E. coli* MG1655 (Foster *et al.* 2013)[1]. Absent from selection the mutations are spaced randomly, with the gap size between adjacent mutations nearly perfectly matching the expected exponential distribution. In contrast, the spacing of mutations identified by QMS-seq shows significant clustering, indicative of selective pressure. The filtered QMS-seq mutations (those analyzed in this study, *see Methods: Filtering mutations under clear selective pressure*) show even greater clustering, suggesting the filtering has successfully discarded most hitchhiker mutations.

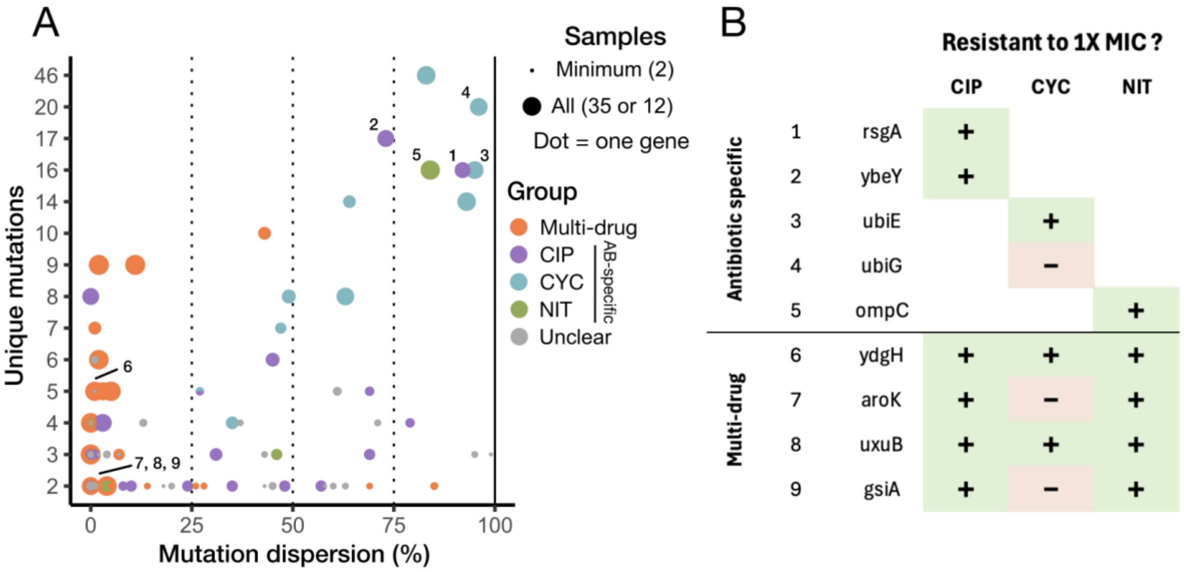

Supplementary Figure 7. Validating resistance of Keio knockout strains

Figure legend: (A) Figure 3A from the main text, annotated to show which genes we picked to validate their role in resistance using the Keio collection. (B) Whether the Keio strain lacking the given gene was resistant to 1X the MIC of the antibiotics that, during QMS-seq, selected for knockout mutations in the same gene.

Sample	Clone	Mutations
CIP/WT_a	1	1
CIP/WT_a	2	1
CIP/WT_b	3	1
CIP/WT_b	4	0
CIP/WT_c	5	0
CIP/WT_c	6	1
CIP/H526Y_a	7	1
CIP/H526Y_a	8	0
CIP/H526Y_b	9	0
CIP/H526Y_b	10	0
CIP/H526Y_c	11	2
CIP/H526Y_c	12	1
CIP/Q513R_a	13	1
CIP/Q513R_a	14	0
CIP/Q513R_b	15	1
CIP/Q513R_b	16	1
CIP/Q513R_c	17	1
CIP/Q513R_c	18	0

Supplementary Table 1. Validating the number of mutations per genome by whole-genome sequencing individual clones.
We chose to examine CIP samples because it leads to DNA damage, and we surmised clones from these samples would have been most likely to acquire additional mutations. Most clones have one mutation per genome, only one was sequenced that had two. Several clones were sequenced with no mutation identified, these likely represent reversions of a high-fitness cost mutation, or cells which survived the antibiotic exposure via transient tolerance mechanisms. Reversions of structural variations (e.g. duplications or transpositions) are especially common, and both are major mechanisms of resistance evolution[2]. Clones with no mutation (and thus no accompanying fitness cost) would also be more prominent because of how we collected colonies to perform individual whole genome sequencing. We picked colonies that had grown over 16 hours after streaking from the resistant heterogeneous populations we sent for metagenomic sequencing. Clones in the heterogeneous population were grown on the plate for three days, as the growth rate of many colonies was very slow. This meant that, after 16 hours, only the most fit clones would be visible on the plate and then picked for sequencing.

sample	numreads	covbases	coverage	meandepth	meanbaseq	meanmapq
CIP/WT_a	52829070	4630065	99.9697	1634.55	35.8	40.8
CIP/WT_b	46440452	4630064	99.9697	1404.29	36	40.8
CIP/WT_c	48638038	4630061	99.9696	1501.82	36	40.8
CIP/H526Y_a	48590278	4630068	99.9698	1508.05	36	40.8
CIP/H526Y_b	51840266	4630062	99.9696	1599.88	35.8	40.8
CIP/H526Y_c	45881192	4630061	99.9696	1404.66	36	40.8
CIP/Q513L_a	77246152	4630062	99.9696	2356.31	36	40.8
CIP/Q513L_b	44335724	4630064	99.9697	1354.55	36	40.9
CIP/Q513L_c	45810618	4630061	99.9696	1440.97	35.9	40.8
CIP/Q513R_a	48772394	4630628	99.9818	1453.03	36.1	40.8
CIP/Q513R_b	40209616	4630061	99.9696	1158.6	36.1	40.6
CIP/Q513R_c	39692048	4630989	99.9896	1186.64	36	40.8
CYC/WT_a	43193514	4630066	99.9697	1330.63	35.7	40.8
CYC/WT_b	50235168	4630212	99.9729	1554.36	36	40.8
CYC/WT_c	37453290	4630065	99.9697	1159.6	36	40.8
CYC/H526Y_a	47740344	4630065	99.9697	1476.49	36	40.8
CYC/H526Y_b	52932154	4630149	99.9715	1575.42	36	40.8
CYC/H526Y_c	59069084	4630061	99.9696	1814.39	36	40.9
CYC/Q513L_a	40291280	4630215	99.9729	1282.53	35.6	40.7
CYC/Q513L_b	52415416	4630599	99.9812	1615.04	35.8	40.8
CYC/Q513L_c	39119024	4630213	99.9729	1246.71	35.7	40.7
CYC/Q513R_a	50847002	4630707	99.9835	1557.38	36	40.9
CYC/Q513R_b	37307404	4630061	99.9696	1155.73	35.9	40.8
CYC/Q513R_c	36743814	4631304	99.9964	1165.1	35.8	40.7
NIT/WT_a	37183914	4630211	99.9728	1178.8	35.7	40.7
NIT/WT_b	41871240	4630062	99.9696	1334.4	35.7	40.7
NIT/WT_c	37862356	4630062	99.9696	1201.19	35.7	40.7
NIT/H526Y_a	47295324	4630065	99.9697	1507.18	35.8	40.7
NIT/H526Y_b	44419324	4630061	99.9696	1412.17	35.7	40.7
NIT/H526Y_c	48362642	4630066	99.9697	1501.08	36	40.8
NIT/Q513L_a	35557340	4630061	99.9696	1132.89	35.8	40.7
NIT/Q513L_b	37858228	4630060	99.9696	1090.41	36.1	40.6
NIT/Q513L_c	42013528	4630062	99.9696	1173.92	36.1	40.4
NIT/Q513R_a	46363924	4630061	99.9696	1435.06	35.9	40.8
NIT/Q513R_b	43951768	4630061	99.9696	1401.17	35.6	40.6

Supplementary Table 2. Sequencing information for each sample.
Sequencing was performed by Azenta Life Science using the Illumina NovaSeq with pair-end 150 bp reads.

Supplementary References

1. Foster, P. L., Hanson, A. J., Lee, H., Popodi, E. M. & Tang, H. On the Mutational Topology of the Bacterial Genome. *G3 GenesGenomesGenetics* **3**, 399–407 (2013).

2. Tomanek, I. *et al.* Gene amplification as a form of population-level gene expression regulation. *Nat. Ecol. Evol.* **4**, 612–625 (2020).
