# Supplementary material for: High-throughput method characterizes hundreds of previously unknown antibiotic resistance mutations
Source: Nat Commun. 2025 Jan 17;16:780. doi: 10.1038/s41467-025-56050-2 (PMC11742677; doi:10.1038/s41467-025-56050-2)
Supplement: Supplementary file 2 — Description of Additional Supplementary Files [file 41467_2025_56050_MOESM2_ESM.pdf]

Description of Additional Supplementary Files

File Name: Supplementary Data 1
Description: All identified mutations

File Name: Supplementary Data 2
Description: All targeted sequences

File Name: Supplementary Data 3
Description: Mobilization events of known insertion sequences

File Name: Supplementary Data 4
Description: Gene ontology terms used for each functional category

File Name: Supplementary Data 5
Description: Geneious file format containing the annotated genome with all identified mutations
